# FIELD WORKERS AT THE INTERFACE

**DOI:** 10.1111/dewb.12027

**Published:** 2013-03-22

**Authors:** Sassy Molyneux, Dorcas Kamuya, Philister Adhiambo Madiega, Tracey Chantler, Vibian Angwenyi, P Wenzel Geissler

This issue of Developing World Bioethics includes a collection of papers on intermediary staff and volunteers working at the interface between research institutions and researchers, and the communities from which research participants are recruited. ‘Field worker’ – a short hand commonly used in many research settings – refers here to those whose main role is face-to-face engagement with participants, who usually speak the participants’ first language, who are from or live in the study areas, and whose work entails moving around the study areas or health facilities. Field workers can be differentiated from medical or scientific staff for whom only part of their duties entail direct interaction with participants, and who are primarily based in the research institution or the clinic. In international research settings field workers are variously called research assistants, community interviewers, data collectors, fieldworkers, field assistants, assessors, follow up staff or defaulter tracers. Although some may hold first degrees or certificates, many are secondary school leavers without higher education opportunity; overall they are formally less qualified than clinical and research staff. Instead, field workers often have extensive informal training and experience from earlier volunteering and jobs in research centres or the NGO sector, which often require similar tasks and expertise.^1^ Their roles may include communicating about studies and mobilisation and follow-up of participants, conducting interviews, and carrying out relatively simple biomedical data-collection procedures such as taking temperatures and collecting finger prick blood samples.

Field workers’ labour relations to research institutions vary. The most clearly formalised is in contracts – in the contexts described by the contributors typically for up to one or two years – which may be extended for periods of up to one or two years, in some cases for decades. At the opposite end of the spectrum we find ‘*volunteers*’, who may be reimbursed expenses, given tokens of appreciation, or payments for specific services. Volunteers may take on fairly similar roles to field staff, including recruitment and follow-up, or data collection, and are sometimes recruited from trial participants in other studies.^2^ Volunteers can also have a more consultative role for example through participating in community advisory boards or other community representative bodies, or through informal links to community based organisations such as youth and womens’ groups or local professional associations (e.g. cycle taxis, sex workers). With most volunteers receiving some income, and with some receiving monthly bank transfers, or even transferring to more stable contractual employment, distinctions between staff and volunteers’ status and duties are not always clear. For our contributors, their key shared characteristic is the interface role of these individuals.

There is growing interest in field staff and volunteers’ role at the interface in international research settings, and in the implications of their activities and challenges for ethical practice. In mediating between the often very different priorities and concerns of well-resourced research institutions, and relatively poor communities without good access to quality affordable health care, field workers are not simply neutrally observing, and adhering to formal, externally derived ethical rules, but instead play ‘a vital, creative, and under-recognised role in research and ethics practice’.^3^

## Challenges in-between

The crucial role that fieldworkers play in facilitating research and ensuring quality data is widely recognised. Reflecting upon the practice of ‘peer recruitment’ to research, Simon and Mosawel (2010, see also commentaries on the paper) draw attention to potential ethical benefits of involving community members in research work: providing monetary benefits to community members who are employed; enhanced research through improved access and responsiveness to local communities; and strengthened consent and information processes that encourage potential participants to ask questions concerning procedures and implications.^4^ On the other hand, Simon and Mosawel also highlight ethical challenges including potential exploitation of staff through unfair employment practices, staff exploiting the trust of peers in their efforts to meet recruitment quotas (including through compromising consent processes), and privacy and confidentiality breaches due to everyday proximity. The latter two concerns were recognised as featuring particularly where community members have prior relationships with potential participants and in cases where recruiters are paid according to performance measures. Similar concerns have been raised by Kombe et al. (2013), discussing instances of scientific misconduct by field staff, through data fabrication or falsification, or through distorting study information to encourage participation.^5^ Often such misconduct occurs in response to real or perceived pressure to meet recruitment targets.^6^

Many of the above practical and ethical issues also apply to those ‘volunteers’ who work in more advisory roles, not directly participating in data-collection or participant contact. Community advisory boards and groups (CAB/Gs) involving volunteers can strengthen research relationships and ethical practice through input to research design and implementation processes. However, documented challenges of CAB/Gs include the selection of members and their representativeness, unclear role definition and inadequate training. The dual role of CAB/Gs as both advancing research and protecting community interests may lead to tensions that are characteristic for the interface position of field workers.

## Field workers in Kenya

The papers in this collection contribute detailed field studies and ethnographic cases from our research sites in western Kenya and the Kenyan coast to the growing body of work on the situated ethics of field workers – employed staff and volunteers – in international medical research.

All papers emphasise the positive role of field workers’ constructive contribution to medical research. Clearly, the close social relations between research staff and the people living in study areas is of mutual benefit to researchers and populations: participants and their communities gain better understandings of research and its procedures, while research institutions benefit from enhanced recruitment and adherence. As described elsewhere, the ‘relational ethics’ – including long term ties and commitments as well as spontaneous responses to particular situations and needs – that regular face-to-face interactions make room for result in enhanced mutual trust and improve collaboration.

The contributors also point out important challenges to field work at the interface between research institutions and populations. Some of these are of a seemingly mundane practical nature, relating to the hardship, even risks, of going ‘to the field’ – which may be a remote village or a poor urban area – entailing long working hours and the vicissitudes of climate and sometimes people. Such perceived challenges are not only important because they may affect job satisfaction and work practices. They also reflect how field workers and other staff conceptualise ‘fieldwork’; in turn potentially influencing attitudes towards research communities and participants.

Many of the challenges of field work described by the papers are inseparable from the positive aspects of close social relations. Thus, several papers mention the challenge to ‘balance one's allegiances’ when working at the interface between groups with different economic and political resources, knowledge and education, and cultures. ‘Dual accountability’, both to the person or institution who pays the worker, and to one's fellow citizens, becomes an issue. This issue of representativeness becomes particularly pertinent in the case of ‘community representatives’ who formally are expected to represent community views and interests in research. Often it is not clear to researchers, community representatives, or community members exactly whose interests the representatives are meant to defend. This question of accountability and identity is also reflected, in concrete fieldwork activities, for example in the dilemmas of HIV follow-up staff when staff conceal that they work for a research organisation when they move around their research areas.

Linked to such issues of identity, field workers face challenges about responsibility and expectations – arising from their relatively privileged economic and sometimes social status vis-a-vis many community members. Across the contributions below, we find this, maybe inevitable, problem. Gikonyo et al.'s ‘fieldworkers’ as formal employees with access to clinicians, and Chantler's ‘village reporters’ as de-facto wage-earners, are faced with expectations of medical and financial assistance in settings where resources are often shared. Kamuya's ‘fieldworkers’ and Adhiambo's ‘follow-up staff’ confront poverty continuously; sometimes giving small amounts of their own money or food to needy participants. Although one might argue that the same challenge of inequality and justice pertains to all levels of international medical research work, field workers face this issue on a particularly personal level.^7^

Such economic considerations are in turn linked to questions of power and influence. Several papers below thus raise the problem that consent, if obtained through good relations, can shade over into persuasion and possibly undue inducement. While good relations indubitably are necessary and valuable to conduct ethically sound and scientifically valid research, there is also a potential for influence and even exploitation, which requires vigilance and reflection.

Yet, as several of the contributions also make clear, the problem of exploitation for field staff in-between research and community also potentially arises in relations between institutions and field workers: when employing relatively less educated staff from within impoverished communities with very few job opportunities. When, on time-limited contracts or volunteering arrangements, there can be concerns about fair staff remuneration and perceived exploitation by the research institution. In connection with this, several papers support the importance, for field workers, of realistic and viable career progression, from local, ground level to more advanced, supervisory roles, through appropriate training pathways.

The definition of fieldworkers’ precise mandate and role, including the overlaps with diverse volunteer roles and the knowledge necessary to perform such roles came up as an issue across several papers. Challenges such as giving adequate information, handling questions, and protecting relationships with fellow community members underline again the importance of systematic training for field workers. Maybe even more importantly, the variegated nature of the challenges described in these case studies – depending on particular social contexts, specific research projects, and sometimes even particular personalities – make it mandatory that collaborative international research institutions provide dedicated systems of supervision and support throughout studies. More broadly, ‘community engagement’ activities aimed at enhancing interactions and mutual understanding between researchers and study communities potentially play an important role in framing and tracking the relationship between field staff and participants, and are themselves complex and contested.

## Studying medical research and its ethics through field workers

In more general terms, the diverse case studies of Kenyan fieldworkers in action brought together by this issue underline the value of fine-grained ethnographic-type and social science research. Such studies can contribute on the one hand to the widening social science literature on contemporary global biosocial assemblages such as international research collaboration, and on the other hand to the work of ethicists and others to safeguard and improve vitally important scientific research within an overall context of global inequality and injustice.
